# Low physical activity is the strongest factor associated with frailty phenotype and frailty index: data from baseline phase of Birjand Longitudinal Aging Study (BLAS)

**DOI:** 10.1186/s12877-022-03135-y

**Published:** 2022-06-10

**Authors:** Ameneh Sobhani, Farshad Sharifi, Reza Fadayevatan, Ahmad Ali Akbari Kamrani, Mitra Moodi, Masoumeh Khorashadizadeh, Toba Kazemi, Huriye Khodabakhshi, Hossein Fakhrzadeh, Masoud Arzaghi, Seyedeh Zahra Badrkhahan, Raziye Sadat Hosseini, Hadi Monji, Amirabbas Nikkhah

**Affiliations:** 1 Department of Gerontology, University of Social Welfare and Rehabilitation Sciences (USWR), Tehran, Iran; 2 Elderly Health Research Center, Endocrinology and Metabolism Population Sciences Institute, Tehran University of Medical Sciences, Tehran, Iran; 3 Iranian Research Center on Ageing, University of Social Welfare and Rehabilitation Sciences (USWR), Tehran, Iran; 4 Ageing Medicine Fellowship, University of Social Welfare & Rehabilitation Sciences, Tehran, Iran; 5 Social Determinants of Health Research Center, Birjand University of Medical Sciences, Birjand, Iran; 6 Cardiovascular Diseases Research Center, Professor of Cardiology, Birjand University of Medical Sciences, Birjand, Iran; 7 Department of Geriatric Medicine, School of Medicine, Tehran University of Medical Sciences, Tehran, Iran; 8 Department of Community Health and Geriatrics, School of Nursing and Midwifery, Iran University of Medical Sciences, Tehran, Iran; 9 School of Medicine, Tehran University of Medical Sciences, Tehran, Iran

**Keywords:** Frailty index, Frailty phenotype, Aged, Prevalence, Low physical activity

## Abstract

**Background:**

Frailty is the most complicated expression of aging that is related to disability or multi-morbidity. The aim of the present study was to estimate the prevalence of frailty and its associated factors among community-dwelling aged population.

**Methods:**

A total of 1529 eligible community- dwelling older adults (≥ 60 years) were enrolled in the baseline phase of Birjand Longitudinal Aging Study (BLAS) from 2019 to 2020. Their frailty status was assessed using the Fried’s frailty phenotype and frailty index. Sociodemographic factors, including sex, age, marital status, and education level, were collected. Health status assessment included the history of hypertension, diabetes mellitus, cardiovascular disease, Alzheimer’s diseases and dementia, and other health conditions. Furthermore, functional assessment (ADL, IADL) and anthropometric measurements including height, weight, waist, calf, and mid-arm circumference were made and the body mass index was calculated. The nutrition status and polypharmacy (use 3 or more medication) were also evaluated.

**Results:**

The prevalence of frailty was 21.69% according to the frailty phenotype and 23.97% according to the frailty index. A multiple logistic regression model showed a strong association between low physical activity and frailty phenotype (OR = 36.31, CI = 16.99–77.56, *P* < 0.01), and frailty index (OR = 15.46, CI = 5.65–42.34, *P* < 0.01). Other factors like old age (≥80), female sex, malnutrition, polypharmacy, obesity, and arthritis were also associated with frailty. The Kappa coefficient of the agreement between these two instruments was 0.18.

**Conclusion:**

It seems that low physical activity is the most important determinant of frailty. Low physical activity and some other factors may be preventable or modifiable and thus serve as clinically relevant targets for intervention.

**Supplementary Information:**

The online version contains supplementary material available at 10.1186/s12877-022-03135-y.

## Background

Frailty is the most complicated expression of aging that is related to disability or multi-morbidity, but it is utterly distinct from them as it is a complex and dynamic process with different aspects of the intervention [1]. Indeed, frailty is a spectrum reflecting the biological age/intrinsic capacity of humans. On the other word, frailty is the end of the “healthy aging” spectrum [1, 2].

It is noteworthy that frailty is an important predictor of multiple adverse outcomes, including disability, hospitalization, institutionalization, premature death, and low resistance to stressors [3]. This means that a similar stressor can have different consequences in a frail individual compared to a robust person [3]. According to observations, the level of frailty is a good predictor or selection criterion for treatment or intervention [4]. Hence, diagnosing frailty in the early stages is critical for preventing or delaying the onset of late-life disability and its adverse outcomes [5].

There are more than 30 assessment tools to measure frailty; however, there is no consensus on a gold standard definition for frailty, which results in differences in estimating the frailty prevalence [6]. The two most frequent tools for diagnosing frailty are the frailty phenotype (FP) and frailty index (FI) [7]. FP only considers physical markers. It is operationalized as a syndrome that includes the five following characteristics: unintentional weight loss, muscle weakness, slow walking speed, low physical activity, and exhaustion [8]. Another approach is the FI, which was developed by Rockwood et al. as a risk index by counting the number of deficits accumulated over time. It includes diseases, physical and cognitive impairments, psychosocial risk factors, and common geriatric syndromes other than frailty [9].

The prevalence of frailty in older adults varies according to the definition, setting, and country. The global prevalence of frailty in population 65 years and older is 10.7% [2]. On the other hand, according to a systematic study in people aged ≥50 years old and in 62 countries, the prevalence of frailty index was 24% (26–22%) and the prevalence of frailty phenotype was 12% (13–11%) [2]. The prevalence of frailty is 12–13.5% in Asia according to the FI instrument and 1.5–18.3% according to the FP instrument [10–14]. Considering the strong association between frailty and other poor health outcomes such as disability, dependency, multi-morbidity, and death, determining the prevalence of frailty and its associated factors in different communities plays an important role in health policy-making [15, 16].

Very few studies have investigated the prevalence of frailty in Iran. A study in Tehran found an age unstandardized prevalence of 60% for frailty. Another study in five cities in the southwest of Iran reported frailty prevalence measured by FI of 14.3% [17, 18]. However, to the best of our knowledge, none of the studies in |Iran compared the prevalence of frailty based on different definitions. Moreover, we could not find any data regarding the prevalence of frailty in rural areas. Therefore, this study was conducted to determine frailty prevalence measured by FP and FI and their associated factors in the community-dwelling older adults (≥ 60) in the rural and urban areas of Birjand county.

## Methods

### Study design and participants

A cross-sectional study was conducted on the baseline data of Birjand Longitudinal Aging Study (BLAS). The BLAS is an ongoing study carried out on a reprehensive sample of community-dwelling older adults ≥60 years living in urban and rural regions of Birjand county to estimate the prevalence of frailty and its associated factors. Birjand is the capital of South Khorasan Province located in the east of Iran [19].

A cohort study was conducted on 1808 older participants in 2018. The participants were selected using multistage stratified cluster random sampling. In the urban regions, according to the postal code, 70 clusters were identified and in each cluster 24 households were selected using the simple random method. The rural participants were enrolled from all ten rural health centers in Birjand county (each health center was located in one village). The inclusion criteria were age ≥ 60 years and the ability to walk (even with assistive instruments). Incomplete data related to frailty and predisposing factors and the data of subjects with severe dementia (AMT < 3) were excluded. Finally, the data of 1529 subjects were analyzed.

**Fig. 1:**
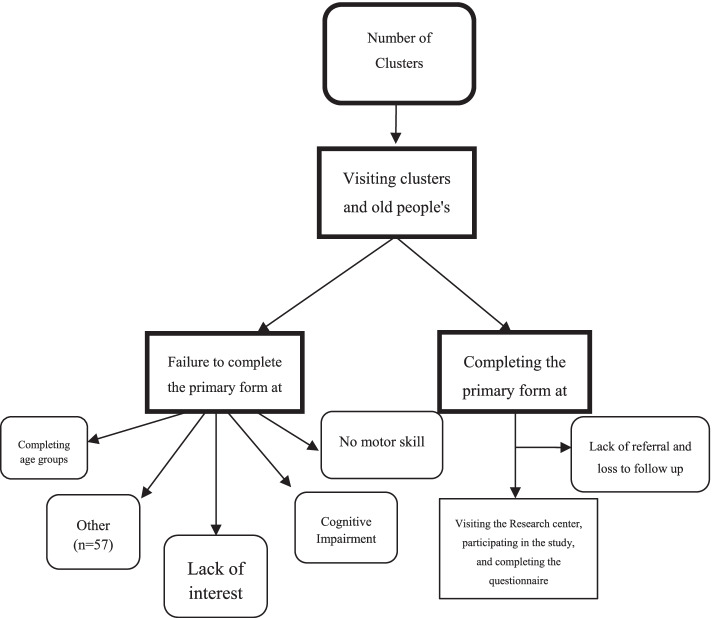
Data collection and sampling

Eight groups of trained researchers gathered the data; each group collected a specific type of data. Figure 1 summarizes the details of data collection and sampling. The data were collected using the Digit software and stored online on a server in Endocrinology and Metabolism Research Institute in Tehran (RABIT: panel.rabit.ir/login.html)). This software can validate the values and helps to prevent missing data. Finally, a response rate of 56.4% was calculated.

Detailed information on study design, data gathering, and measurements in BLAS was described previously [19].

### Variables

#### Sociodemographic and health status assessment

Comprehensive questionnaires were used to collect sociodemographic and education status, years of classic schooling, occupation, etc.). The history of chronic diseases was also inquired from the subjects or their companions (e.g., hypertension, diabetes mellitus, cardiovascular diseases, Alzheimer’s and dementia, Parkinson’s diseases, osteoporosis, osteoarthritis, hyperlipidemia, heart failure, cancers, gout, thyroid disorders, liver and kidney diseases, depression, and other psychiatric disorders). The history of other health deficits comprising the frailty index was collected too. Furthermore, drug history in the past six months was also recorded (prescription and over-the-counter medication) through asking relevant questions from the participants and their companions. The participants’ medical documents were also evaluated. The participants were grouped into two clusters: those who used three medications and more and those who used less than three medications.

#### Frailty phenotype (FP)

The FP criteria were extracted from the Cardiovascular Health Study [8]. FP has five components, including weight loss (> 10 lbs. lost unintentionally over a one-year period), low walking speed (walking time for 15 ft: the slowest 20% [by sex and height]), low handgrip strength (the lowest 20% [by sex and BMI]), exhaustion (self-report) and low physical activity levels (kcals/week: males; < 383 kcals/week males: and females; < 270 kcals/week) [8].

The duration of walking a distance of 4.57 m at a maximum speed was recorded three times. The handgrip force of both hands was also measured three times in sitting position with an arm-forearm angle of 90° by an electronic hand dynamometer (Saehan, Seoul, Korea) calibrated before starting of measurements. The interval between two measurements in each hand was at least 2 min. Timed get up and go (TUG) test was also performed according to the standard method using a chair without a handle. The participants were asked to get up from the chair, walk three meters, then go back at their usual speed, and finally sit again on the chair. The time spent on this process was measured and recorded.

Physical activity was measured using Longitudinal Aging Study Amsterdam Physical Activity Questionnaire (LAPAQ). This questionnaire assesses the physical activity of community-dwelling older adults at work and during leisure time [20]. According to the table for calculation of metabolic equivalence (METs), the METs /min of physical activity was calculated and the 20% lowest METs/min by sex was considered as a low physical activity level. Low physical activity was defined according to the World Health Organization (WHO) definition (physical activity < 300 MET/min was considered as low physical activity as an independent variable in models).

Each item in the frailty phenotype scored one point. The participants were defined as robust if their score was zero out of five, pre-frail if they met one or two criteria, and frail if they scored three or more.

#### Frailty index (FI)

FI was defined based on a cumulative approach to impairments and illnesses collectively known as deficits including disability, diseases, physical and cognitive disorders, psychosocial risk factors, and geriatric syndromes (e.g., falls, delirium, and urinary incontinence). The presence of each condition was coded as one and absence of each one was coded as zero. The FI was constructed using a standardized procedure[21] and totally, 29 age-related health deficits were included to calculate the FI [9]. The FI score was defined as the ratio between the existing deficits and the total number of evaluated deficits. Thus, the FI ranged from zero to one (zero indicating no deficits and one indicating the presence of all deficits). To detect the severity of frailty and the agreement between classifications, FI, which is a continuous score, was categorized as robust (0 to < 0.20), pre-frail (0.20 to 0.25), and frail (> 0.25) [22].

#### Functional assessment

The Barthel’s index and Lawton tool were used to assess activities of daily living and instrumental activities of daily living, respectively. Barthel’s index has 10 items with a maximum score of 20 for totally independent and 0 for totally dependent subjects on all items [23]. The total score of the Lawton Instrumental Activities of Daily Living ranges between 8 and 0. This tool assesses the independence of the subject in performing eight domains of intermediate daily function (ability to use telephone, shopping, food preparation, housekeeping, laundry, taking responsibility for own medications, handling finances, and mode of transportation) [24].

#### Anthropometric measurements

Anthropometric indices were measured according to the National Health and Nutrition Examination Survey anthropometry procedures manual [25]. Trained researchers measured height with minimal clothing without any head cover to the nearest 0.1 cm while four points of the body (back of the head, back of shoulder buttock, and heal) touched the behind wall and the head was in a neutral position. Weight was measured to nearest 0.1 kg with minimal clothing using a calibrated SECA digital scale (SECA, Germany). Waist circumference was measured at the iliac crest with a non-elastic tape held horizontally after an overnight fast. The calf circumference was measured bilaterally at the maximum circumference with the knee flexed at 90°. Moreover, the mid-arm circumference was measured at the middle distance between the cricoid and olecranon processes. Body mass index (BMI) was calculated as weight (kg) divided by square of height (meter). Then, the participants were categorized into four groups according to the World Health Organization (WHO) definition (< 18.5 kg/m2 = low body weight, 18.5–24.99 kg/m2 = ideal body weight 25–29.99 kg/m2 = overweight, and ≥ 30 kg/m2 = obese).

#### Nutritional assessment

Mini Nutritional Assessment (MNA) was used to assess the nutritional status of the participants. It is an 18-item questionnaire on anthropometry measurements, diet, and global assessment in two subgroups with a maximum score of 30. The participants were divided into three groups, including malnourished (score ≤ 16.5), at risk of malnutrition (17 ≤ score ≤ 23.5), and well-nourished (score > 23.5). This tool is one of the most applicable nutritional screening instruments among older adults validated in the Iranian population [26–28].

### Statistical analysis

A survey analysis weighted to Birjand county’s older population was used (number of subjects in age groups [60–69 years, 70–79 years, 80+ years] in Birjand county divided by number of subjects in each age group in our sample were considered as weight of population). We performed direct age-standardization the prevalence rates according to the standard WHO population 2000–2025. The association between FI and FP as dummy variables with other factors was evaluated using univariate and multiple logistic regression models. Backward multiple logistic analysis was carried out and the variables with *P* ≤ 0.10 remained in the model. Final model for frailty phenotype contained age group (60–69 years, 70–79 years, 80+ years), nutritional status according to MNA (malnourished; MNA < 17, at risk of malnutrition; 17 ≤ MNA ≤ 23.5, and well-nourished; MNA ≥ 24), low physical activity; < 300 METs/min in week, gender, and osteoarthritis. For frailty index the final model included age group (60–69 years, 70–79 years, 80+ years), nutritional status according to MNA (malnourished; MNA < 17, at risk of malnutrition; 17 ≤ MNA ≤ 23.5, and well-nourished; MNA ≥ 24), low physical activity; < 300 METs/min in week, gender, polypharmacy (consumption of 3 medication or more), and BMI. We performed a sensitivity analysis for association between low physical activity and FP by removing of low physical activity from components of FP and then we ran again the model. For removed effect of multimorbidity on association of FI and polypharmacy, we considered also multimorbidity in model and assessed the independent association between polypharmacy an FI. The Stata software version 12 (College Station, TX, USA) was used for data analysis.

## Result

### Sample characteristics

A total of 1529 subjects aged 60 years and above were included in this study. The mean age of the participants was 70.6 years (range: 60–97 years) with a standard deviation (SD) of 8.2 years. In total, 789 (51.6%) subjects were women, 1224 (80%) were married, 471 (30.8%) had primary school education, and 520 (34%) reported polypharmacy (Table 1).

**Table 1:** Characteristics of participants according to their frailty phenotype category

		Robust		Pre-Frail		Frail		Total		*P*-Value
		Freq.	percent	Freq.	percent	Freq.	percent	Freq.	percent	
Sex	Female	98	40.5	613	53.7	78	53.4	789	51.6	< 0.01
	Male	144	59.5	528	46.2	68	46.5	740	48.4	
Age	60–69	198	81.8	614	53.8	47	32.1	859	56.1	< 0.01
	70–79	36	14.8	385	33.7	48	32.8	469	30.6	
	≥80	8	3.3	142	12.4	51	34.9	201	13.1	
Marital Status	married	218	90	904	79.2	102	69.8	1224	80	< 0.01
	single	1	0.4	8	0.7	1	0.6	10	0.6	
	widow	18	7.4	217	19	42	28.7	277	18.1	
	separated	5	2	12	1	1	0.6	18	1.1	
Education level	illiterate	71	29.3	570	49.9	98	67.1	739	48.3	< 0.01
	Primary school	71	29.3	363	31.8	37	25.3	471	30.8	
	High school	19	7.8	56	4.9	6	4.1	81	5.3	
	diploma	40	16.5	88	7.7	4	2.7	132	8.6	
	academic	41	16.9	64	5.6	1	0.6	106	6.9	
BMI	Low body weight	11	4.5	53	4.6	10	6.8	74	4.8	< 0.05
	Ideal body weight	106	43.8	407	35.6	68	46.5	581	38	
	Over weight	93	38.4	434	38	42	28.7	569	37.2	
	Obese	32	13.2	247	21.6	26	17.8	305	19.9	
Physical activity	high	242	100	1050	92	87	59.6	1379	90.1	< 0.01
	low	0	0	91	7.9	59	40.4	150	9.8	
Smoking	No	222	91.7	1066	93.4	137	93.8	1425	93.2	0.60
	Yes	20	8.2	75	6.5	9	6.1	104	6.8	
Stroke	No	234	96.7	1091	95.6	133	91.1	1458	95.3	0.02
	Yes	8	3.3	50	4.3	13	8.9	71	4.6	
DM	No	196	80.9	853	74.7	112	76.7	1161	75.9	0.11
	Yes	46	19	288	25.2	34	23.3	368	24	
Cancer	No	242	100	1133	99.3	145	99.3	1520	99.4	0.42
	Yes	0	0	8	0.7	1	0.6	9	0.6	
Arthritis	No	208	85.9	940	82.3	108	73.9	1256	82.1	0.01
	Yes	34	14	201	17.6	38	26	273	17.8	
HTN	No	155	64	650	56.9	80	54.8	885	57.8	0.09
	Yes	87	35.9	491	43	66	45.2	644	42.1	
Respiratory	No	221	90.8	1020	89.4	123	84.1	1367	89.1	0.24
	Yes	21	8.6	121	10.6	23	15.7	165	10.8	
CKD	NO	237	97.9	1107	97	141	96.5	1485	97.1	0.68
	Yes	5	2	34	2.9	5	3.4	44	2.8	
Digestive problem	NO	200	82.6	913	79.6	98	67.1	1211	79.2	< 0.04
	Yes	42	17.3	228	19.9	48	32.8	318	20.8	
Swallowing problem	No	240	99.1	1114	97.6	133	91	1486	97.2	< 0.01
	Yes	2	0.8	27	2.3	13	8.9	42	2.7	
MNA total	Malnourished	1	0.4	14	1.2	4	2.7	19	1.2	< 0.01
	At risk	29	11.9	277	24.2	73	50	379	24.8	
	Well nourished	212	87.6	850	74.5	69	47.2	1131	73.9	
Use more 3 drugs	No	179	73.9	753	65.9	77	52.7	1009	65.9	< 0.01
	yes	63	26	388	34	69	47.2	520	34	

### Prevalence of frailty

The frailty status was determined using FI and FP. The crude prevalence of frailty was 9.55% according to the FP criteria versus 11.12% using the FI criteria (Table 2). The WHO age-standardized frailty prevalence according to FP was 21.7% in all participants, 29.8% in adults ≥80 years, 23.7% in women, 22.5% in urban regions, and 24.5% in individuals with low body weight (Table 3).

**Table 2:** Crude prevalence of frailty phenotype and frailty index

	Frailty Phenotype		Frailty Index	
	Frequency	Percent	Frequency	Percent
Robust	242	15.8	467	30.5
Pre frail	1141	74.6	892	58.3
Frail	146	9.5	170	11.1

**Table 3:** Standardized prevalence of frailty phenotype

			Robust		Pre-Frail		Frail	
			Proportion %	95% CI	Proportion %	95% CI	Proportion %	95% CI
Total participants			6.7	5.4–8.3%	71.5	66.5–76%	21.6	17.4–26.6%
	Sex	Female	4.4	3.1–6.2%	71.8	64.2–78.4%	23.6	17.3–31.4%
		Male	9.2	7–12%	71.3	65–76.8%	19.4	14.3–25.6%
	Age groups	60–69	23.1	20.3–26.2%	71.4	68.2–74.4%	5.3	4–7.1%
		70–79	7.4	5.2–10.4%	81.4	77.3–84.9%	11	8.2–14.6%
		+ 80	2.7	1.3–5.5%	67.4	59.3–74.5%	29.8	22.8–37.8%
	Age groups female	F (60–69)	17.5	14.4–21.2%	76.5	72.5–80.1%	5.8	4.1–8.4%
		F (70–79)	4	2.1–7.6%	84.5	79–88.8%	11.3	7.6–16.4%
		F (+ 80)	1.5	0.03–5.9%	65.5	53.3–76%	32.8	22.6–45%
	Age groups male	(60–69)	29.1	24.6–33.9%	66.1	61.1–70.8%	4.7	2.9–7.5%
		M (70–79)	10.7	7.1–15.9%	78.4	72–83.6%	10.7	7–16.1%
		M (+ 80)	4.1	1.7–9.5%	69.5	59.5–78%	26.3	18.3–36.2%
	Living area	Urban region	6.5	5.2–8.1%	70.9	65.5–75.7%	22.5	17.9–27.9%
		Rural region	8.6	4.9–14.7%	75.7	62.2–85.4%	15.6	7.4–29.9%
	WHO BMI categorization	Low body weight	7.2	3.1–15.7%	68.2	51.8–81.1%	24.5	13.1–40.9%
		Ideal body weight	8	6.1–10.3%	70.3	63.7–76.2%	21.6	16.1–28.3%
		Overweight	7.7	5.2–11.4%	71	60–80%	21.1	12.9–32.5%
		Obese	2.8	1.7–4.5%	76.9	58.7–88.6%	20.2	9.1–39.1%

*CI* confidence interval, *BMI* body mass index

Note: Standardized for WHO population 2000-2025

The standardized frailty prevalence according to FI was 23.9% in all participants, 34.9% in women, 31.6% in adults ≥80 years, 26.3% in urban regions, and 42.8% in obese people (Table 4).

**Table 4:** Standardized prevalence of frailty index

			Robust		Pre-Frail		Frail	
			Proportion %	95% CI	Proportion %	95% CI	Proportion %	95% CI
Total participants			18.8	16.1–21.8%	57.1	52.1–62.1%	23.9	19.5–29%
	Sex	Female	4.8	3.5–6.6%	60.2	52.6–67.4%	34.9	27.9–42.6%
		Male	33.4	28.3–38.9%	54.1	47.7–60.3%	12.4	8.2–18.3%
	Age groups	60–69	36.7	33.4–40%	58	54.6–61.4%	5.2	3.9–6.9%
		70–79	25.4	21.4–29.8%	58.7	53.9–63.4%	15.8	12.5–19.7%
		+ 80	12	8.4–16.9%	56.3	48.3–64%	31.6	24.4–39.7%
	Age groups female	F (60–69)	19.9	16.6–23.7%	70.4	66.2–74.4%	9.6	7.2–12.6%
		F (70–79)	4.4	2.4–7.9%	69	62.3–75%	26.5	20.9–33%
		F (+ 80)	1.5	0.4–5.9%	54.3	42.4–65.7%	44.1	32.7–56.1%
	Age groups male	M (60–69)	54.3	49.1–59.4%	44.9	39.9–50.1%	0.6	0.1–2.6%
		M (70–79)	45.7	39–52.6%	48.8	41.9–55.7%	5.4	2.8–10%
		M (+ 80)	23.5	16.4–32.6%	58.3	48.2–67.8%	1.8	11.3–27.5%
	Living area	Urban region	18.1	15.4–21.2%	55.4	50–60.7%	26.3	21.5–31.8%
		Rural region	23.2	15.3–33.5%	70.5	58.5–80.1%	6.2	1–18.3%
	WHO BMI categorization	Low body weight	24.2	15–36.5%	58.5	42.3–73.1%	17.2	7.7–34.2%
		Ideal body weight	20.9	17.2–25.2%	58.7	52–65.1%	20.2	14.8–27%
		Overweight	20.7	15.3–27.3%	56.4	45.9–66.3%	22.9	14.6–33.9%
		Obese	7.2	5.2–9.9%	49.9	34.5–65.2%	42.8	28.2–58.8%

*CI* confidence interval, *BMI* body mass index.

Note: Standardized for WHO population 2000-2025

**Fig. 2:**
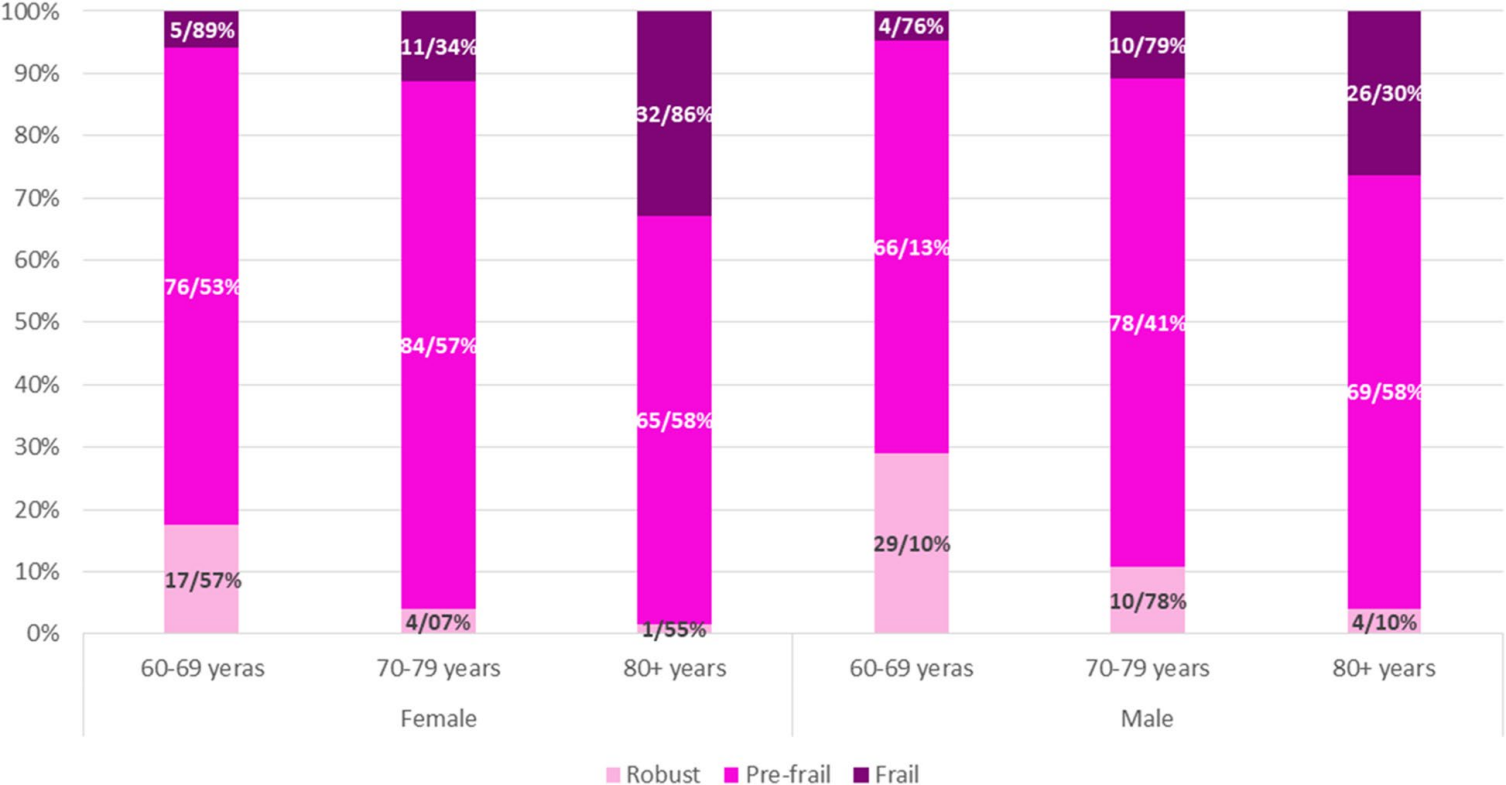
The prevalence of frailty phenotype in men and women in three age groups

**Fig. 3:**
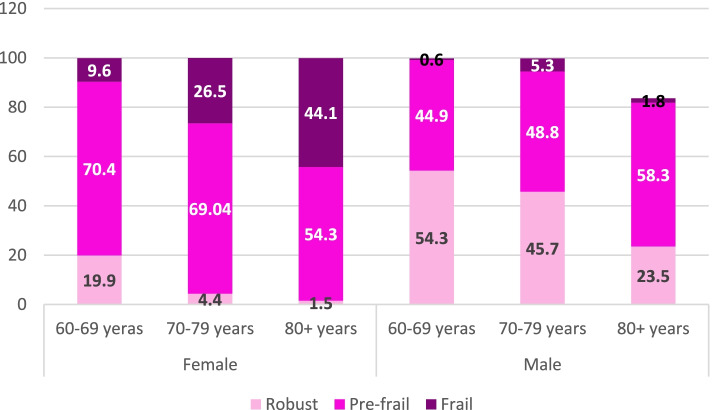
The prevalence of frailty index in men and women in three age groups

Figures 2 & 3 demonstrated the frailty prevalence measured by FP and FI in men and women in three age group.

### Factors associated with frailty

The relationships between FP and FI and other variables ware investigated. In a logistic multiple models, a strong association was found between low physical activity and FP (OR = 36.3, CI = 16.9–77.5, *P* < 0.01), and FI (OR = 15.4, CI = 5.6–42.3, *P* < 0.01). Other factors like advanced age (≥80) (FP (OR = 5.6, CI = 3.4–9.2, *P* < 0.01), FI (OR = 9.7, CI = 5.8–16.1, *P* < 0.01) and female sex (FP (OR = 0.1, CI = 0–0.3, *P* < 0.01), FI (OR = 0.04, CI = 0.01–0.1, *P* < 0.01) were associated with frailty according to both instruments. Obesity (OR = 3.5, CI = 1.2–10.5, *P* = 0.02), polypharmacy (OR = 2, CI = 1.4–3, *P* < 0.01), and malnutrition (OR = 0.3, CI = 0–1, *P* < 0.01) were associated only with FI, while arthritis was associated with FP in older adults (OR = 1.6, CI = 1–2.6, *P* < 0.02) (Tables 5 and 6).

**Table 5:** The univariate and multivariate analyses for the associated factors and frailty phenotype

		OR	95% CI	*P*-Value
**Univariate**				
Age Group	60–69	1		
	70–79	1.96	1.29–2.99	< 0.02
	+ 80	5.87	3.81–9.05	< 0.01
MNA-cat	Malnourished	1		
	At risk of malnutrition	0.89	0.28–2.77	0.8
	Well nourished	0.24	0.07–0.75	0.01
Low physical activity		9.62	6.49–14.26	< 0.01
Gender (male/female)		0.92	0.65–1.29	< 0.01
Poly pharmacy (use 3 or more medication)		1.85	1.31–2.6	< 0.01
Chronic diseases-FI		1.34	1.01–1.78	0.04
Smoking		0.89	0.43–1.8	0.7
BMI	Low body weight	1		
	Ideal body weight	0.84	0.41–1.73	0.6
	Overweight	0.51	0.24–1.06	0.07
	Obese	0.59	0.27–1.29	0.1
Waist		0.98	0.96–0.99	0.01
Diabetes		0.95	0.63–1.42	0.8
HTN		1.14	0.81–1.61	0.4
CHF		1.15	0.82–1.62	0.3
COPD		1.35	0.3–6.03	0.6
Cancer		1.18	0.14–9.54	0.8
Arthritis		1.71	1.15–2.55	< 0.07
**Multivariate**				
Age Group	60–69	1		
	70–79	1.83	1.16–2.87	< 0.09
	+ 80	5.62	3.42–9.22	< 0.01
MNA-cat	Malnourished	1		
	At risk of malnutrition	1.03	0.28–3.76	0.9
	Well nourished	0.41	0.11–1.52	0.1
Low physical activity		36.31	16.99–77.56	< 0.01
Gender (male/female)		0.15	0.07–0.32	< 0.01
Arthritis		1.66	1.05–2.62	0.02

**Table 6:** The univariate and multivariate analyses for the associated factors and frailty index

		OR	95% CI	*P*-Value
**Univariate**				
Age Group	60–69	1		
	70–79	2.81	1.9–4.15	< 0.01
	+ 80	6.36	4.15–9.73	<0.01
MNA-cat	Malnourished	1		
	At risk of malnutrition	0.47	0.1–1.24	0.1
	Well nourished	0.13	0.5–0.34	<0.01
Low physical activity		1.7	1.07–2.7	0.02
Gender (male/female)		0.19	0.13–0.29	<0.01
Poly pharmacy (use 3 or more medication)		3.19	2.3–4.42	<0.01
Smoking		0.14	0.03–0.6	<0.08
Waist		1.01	1–1.03	0.01
BMI	Low body weight	1		
	Ideal body weight	1.5	0.6–4.02	0.3
	Overweight	1.44	0.55–3.74	0.4
	Obese	2.83	1.09–7.37	0.03
**Multivariate**				
Age Group	60–69	1		
	70–79	3.21	2.1–4.9	<0.01
	+ 80	9.71	5.85–16.14	<0.01
MNA-cat	Malnourished	1		
	At risk of malnutrition	0.66	0.2–2.15	0.4
	Well nourished	0.32	0.09–1.04	0.05
Low physical activity		15.46	5.65–42.34	<0.01
Gender (male/female)		0.04	0.01–0.1	<0.01
Poly pharmacy (use 3 or more medication)		2.06	1.41–3.02	<0.01
BMI	Low body weight	1		
	Ideal body weight	1.88	0.66–5.34	0.2
	Overweight	2.03	0.7–5.89	0.18
	Obese	3.58	1.21–10.55	0.02

### Sensitivity analysis

To remove the effect of low physical activity in composing of frailty phenotype on our model, we defined another time the frailty phenotype without including of low physical activity and then other time runed the model the association between of new defied PF and physical activity became more stronger (odds ratio of physical activity = 36.31 95% CI (17.00–77.56) (Table 2 Supplementary).

Furthermore, we included the multimorbidity in our model for assessing the independent effect of polypharmacy on FI and the polypharmacy significantly related to FI index (Table 2 Supplementary).

### Agreement between FI and FP

The agreement between groups was assessed using the Kappa coefficient. The Kappa coefficient between these categories was 0.18.

## Discussion

The present study was conducted in an adult population aged ≥60 years in one of provincial capitals in the east of Iran. The results showed a high age-standardized prevalence of frailty phenotype and frailty index in this part of Iran. low physical activity was one of the most important factors with a strong positive relationship with frailty, and FP was about 36.5 times more prevalent in the group with low physical activity and the age- standardized prevalence of FI was about 15.5 times higher) compared to the group with moderate to high physical activity.

The prevalence of frailty in the present study was consistent with other studies in Iran [17, 29], and other countries such as Japan [10], Singapore [11], and Thailand [12]. However, a study by Abdi et al. found a frailty prevalence of 60% in the older adult population of Tehran, Iran [18]. One of the reasons for this discrepancy may be the use of different instruments, as Abdi et al. used the Tilburg Frailty Indicator [18] and we used Frailty Phenotype and Frailty Index. Another reason could be that they did not perform age-standardization. However, the results of the present study and the above studies indicate the high prevalence of frailty in Iran.

This study is one of first studies the compared the frailty phenotype and frailty index prevalence in community living older adults in Iran. Moreover, this study indicated a very strong independent association between FP also FI and low physical activity even when low physical activity was not considered as a component of FP.

Lack of physical activity was considered as the most important risk factor for frailty, especially frailty phenotype, which was in line with other studies [11, 29–31]. Physical activity is one of the frailty phenotype components, which was expected to be associated with frailty. However, this association was too strong to consider low physical activity as a mere component of FP. High levels of physical activity may have a role in controlling pathophysiological pathways of frailty development. Moreover, resistance training helps to increase the mass and strength of the muscles as well as the physical performance [32]. On the one hand, studies have shown that moderate and high levels of physical activity have an important role in decreasing the serum levels of inflammatory factors such as interleukin 6, interleukin 10, tumor necrotizing factors α, and C-reactive protein [33, 34]. Furthermore, the role of inflammatory processes in frailty development becomes more and more clear every day [35].

According to the results, about 50% of the subjects with frailty were over 80 years old, which is consistent with other studies [36–39], indicating that the prevalence of frailty increases with age [37, 40, 41]. The effect of aging on frailty may be associated with a reduction in the physiological reserve during the life [42] as well as additional age-associated pathological changes [8]. Although aging is a risk factor for frailty, all older adults are not frail [43], suggesting that the development of frailty needs additional drives for expanding this condition than the process of aging [44].

Frailty defined by both FI and FP were much more common among women than in men in the present study. Several studies have reported similar results [10, 12, 14, 29, 40, 46–48]. A study in Mexico found that frailty was more common in women using the FI definition; however, frailty was more common in men based on the FP instrument [45]. In addition, two studies conducted in Canada and Singapore found no relationship between gender and prevalence of frailty [46, 47]. However, some epidemiological studies have shown that women are may more likely to be frail at any age using any frailty definition tool [10, 12, 14, 29, 40, 46–48, 51]. Because women have a higher physiological reserve, which helps them tolerate several chronic diseases in multiple organ systems (i.e., greater morbidity) and have a longer lifespan [48]. Older women have more abdominal fat than men, which may play an important role in increasing inflammatory factors [49] and developing frailty [50]. Despite more social support, women are more socially vulnerable than men due to life events (widowhood and loneliness) [48]. However, their better social support will help them manage higher frailty levels compared to men [48].

The study of the prevalence of frailty in urban and rural areas of 8 countries (Cuba, Dominican Republic, Puerto Rico, Venezuela, Peru, Mexico, China, and India) with different cultures and lifestyles showed that in all areas the prevalence of frailty by FP was reported more in women than men [51].

One study has reported that men were more likely to die suddenly than women, while women experienced a steady decline [52]. This reduction has the potential to lead to vulnerability and provide a clearer picture of frailty for women. Another possible explanation is that women have more life expectancy and as a result experience lower quality of life and poorer health status in the last years of their lives [53]..

The FI instrument indicated that polypharmacy was associated with higher frailty, which is in agreement with previous studies [11, 14, 54]. In a study of 4402 participants with a mean age of 61.2 years and a follow-up time of 8 years, Veronese et al. found that the incidence of frailty was nearly two-fold higher in those who used 4–6 medications and six-fold higher people who used more than seven medications [55]. Some reasons should be considered to understand the relationship between polypharmacy and frailty. First, polypharmacy may have an adverse effect on the conditions related to frailty (such as comorbidities) or elements involved in frailty definitions such as weight loss. The intake of several medication can be related to wrong prescription, low adherence, and unnecessary hospitalization, altogether contributing to frailty [55].

The present study confirmed the hypothesis that obese (higher BMI) older adults are more likely to be frail. Obesity plays an important role in the development of frailty and is a significant risk factor for many adverse health outcomes, disabilities, and comorbidities associated with frailty [40, 56–58]. In 4019 participants aged 41–81 years from the Doetinchem Cohort Study, a U-shaped association was found between BMI and frailty, where those with low and high BMI had higher levels of frailty [59]. Previous studies suggest that inflammation may play a critical role in the relationship between obesity and frailty [56]. The older adult subjects with frailty usually have high levels of inflammatory markers such as C-reactive protein, interleukin-6, and fibrinogen [56]. On the other hand, it has been recently recognized that the majority of the obese older adults also meet criteria for frailty because of decreased muscle mass and strength that occurs with aging (sarcopenia) and a need to carry a greater body mass due to obesity [33].

The prevalence of frailty in urban areas was higher than in rural areas according to both instruments. Cakmur conducted a study in rural areas of Turkey [60], that estimated the prevalence of frailty based on the FP instrument as 7%, which can be attributed to the higher physical activity of the rural population, group living in these areas, and better economic status of individuals [60].

According to the study in China, the prevalence of frailty was reported to be higher in urban areas (9.1%) than in rural areas (5.4%) by FP [51]. Another study in the Japanese population showed that the prevalence of frailty in the city and suburbs is higher than the rural population [61]. A systematic review and meta-analysis of the prevalence of vulnerability in China confirms the higher prevalence of frailty in urban areas than in rural areas [10].

 The higher prevalence of frailty in urban areas may be justified significantly by lower socioeconomic status in these areas due to urbanization and machine life, nutritional status or social activity. The people in urban areas also may be less likely to be underweight and are more likely to be overweight and have high blood pressure, diabetes, bone and joint disease [61]. All of these finding were true in our study, but the results were not shown in this article.

There is a growing need to fill the gap of knowledge concerning frailty and its consequences. By contrast, factors associated with frailty are not clearly identified, which would make it challenging to design primary preventative measures [45].

The main limitation of this study was that very frail adults and those who were unable to communicate or move could not participate in our study. It should be noted that our approach in this study was to assess cross-sectional relationships, which makes it impossible to draw causal interferences. Another limitation was that we assessed physical activity by a questionnaire. Assessing of the physical activity by questionnaires may not be very precise, particularly in older adults, because they may not report their level of physical activities accurately, so it can be affected the result.

## Conclusion

The authors believe that investigations aiming at frailty syndrome and its associated factors, especially low physical activity, can be the cornerstone of designing effective interventions to prevent poor conditions and improve the health of the older adults. The present study found that physical activity had a stronger association with frailty in older adults. Previous studies showed that the factors associated with frailty syndrome varied in different geographical locations and cultures. Therefore, in order to design an effective intervention program, it was necessary to conduct a study on the Iranian older adult population.

## Supplementary Information

**Additional file 1.**

## Data Availability

All data used and analyzed in this study are not available. (We cannot publish public data because Tehran University of Medical Sciences and Birjand University of Medical Sciences, which own the data, do not agree with publishing the data, but if the editors of the journal or someone want it privately should be contacted to Farshad Sharifi; first responsible author.)

## Abbreviations

FI: Frailty index
FP: Frailty phenotype
BLAS: Birjand longitudinal aging study
BMI: Body mass index
LAPAQ: Longitudinal aging study Amsterdam physical activity questionnaire
MET: Metabolic equivalence
WHO: World health organization
ADL: Activities of daily living
IADL: Instrumental activities of daily living
MNA: Mini nutritional assessment
AMT: Abbreviated mental test
