# Supplementary material for: Low physical activity is the strongest factor associated with frailty phenotype and frailty index: data from baseline phase of Birjand Longitudinal Aging Study (BLAS)
Source: BMC Geriatr. 2022 Jun 10;22:498. doi: 10.1186/s12877-022-03135-y (PMC9188172; doi:10.1186/s12877-022-03135-y)
Supplement: Supplementary file 1 — Additional file 1. [file 12877_2022_3135_MOESM1_ESM.docx]

Table 1 supp: Frailty Index variables

|  | **Variable** | **Definition of variable** |
| --- | --- | --- |
| 1 | Lawton’s transporting | 0= Travels independently on public transportation or drives own car  0 = Arranges own travel via taxi, but does not  otherwise use public transportation  0= Travels on public transportation when  accompanied by another  0= Travel limited to taxi or automobile with  assistance of another  1= Does not travel at all |
| 2 | Barthel’s dressing | 1= dependent  0 = needs help, but can do about half unaided  0= independent (including buttons, zips, laces etc) |
| 3 | Barthel’s stairing | 1= unable  0= needs help (verbal, physical, carrying aid)  0= independent up and down |
| 4 | Time Get Up & Go | 5 times get up time:  1= cannot do it  0.75= more than 12.9 s  0.5= 9.4-12.9 s  0.25= 6.97-9.4 s  0= 6.97 s |
| 5 | Full tandem standing | Standing with fulltandom  0= Standing for 10 s  0.5= < 10 s  1= Cannot do it |
| 6 | How many times did you walk during the past two weeks? | 1= 0  0.75= 1-5  0.5= 5-12  0.25= 12-14  0= 14-30 |
| 7 | Low physical activity | 0= >= 300 METs minus per week  1= less than 300 METs minutes per week |
| 8 | MMSE (orient time) | 0= 5 0.5= 4 1= 0-3 |
| 9 | MMSE (orient place) | 0= 5 0.5= 4 1= 0-3 |
| 10 | MMSE (attention) | 0= 5 0.5= 3-4 1= 0-2 |
| 11 | MMSE (recall) | 0= 3 0.5= 2 1= 0-1 |
| 12 | Gait speed 6m | 0 ≥0.8 m/s 1<0.8 m/s |
| 13 | General health | 0= no 1= yes |
| 14 | Feel depressed | 0= no 1= yes |
| 15 | Feel effort | 0= no 1= yes |
| 16 | Enjoy life | 0= no 1= yes |
| 17 | Not can | 0= no 1= yes |
| 18 | Stork | 0= no 1= yes |
| 19 | DM | 0= no 1= yes |
| 20 | Cancer | 0= no 1= yes |
| 21 | Arthritis | 0= no 1= yes |
| 22 | HTN | 0= no 1= yes |
| 23 | Memory | 0= no 1= yes |
| 24 | Cardiac | 0= no 1= yes |
| 25 | Feel energetic | 0= no 1= yes |
| 26 | PAD | 0= no 1= yes |
| 27 | Lung disease | 0= no 1= yes |
| 28 | Other chronic disease | 0= no 1= yes |
| 29 | Incontinence | 0= no 1= yes |

Table 2 supp: The association between FP and low physical activity (excluded physical activity form components of FP)

|  | | OR | 95% CI | P Value |
| --- | --- | --- | --- | --- |
| Age Groups | 60 - 69 | 1 |  |  |
|  | 70 - 79 | 1.94 | 1.37 – 2.74 | <0.01 |
|  | + 80 | 6.73 | 4.52 – 10.02 | <0.01 |
| MNA | Malnourished | 1 |  |  |
|  | At risk of malnutrition | 0.63 | 0.22 – 1.83 | 0.4 |
|  | Well nourished | 0.33 | 0.11 – 0.95 | 0.04 |
| Low physical activity | | 3.79 | 2.22 – 6.46 | <0.01 |
| Gender (male/female) | | 0.24 | 0.16– 0.36 | <0.01 |
| Arthritis | | 1.4 | 0.97 - 2 | 0.06 |

FP: frailty phenotype, OR: odds ratio, CI: confidence interval

Table 3 supp.: The association between FI and low physical activity (excluded low physical activity from components of FI

|  |  | Odds ratio | 95% CI OR | | P Value |
| --- | --- | --- | --- | --- | --- |
| Age groups | 60-70 years | 1 |  |  |  |
|  | 70-79 | 3.03 | 1.92 | 4.78 | <0.01 |
|  | 80+ | 10.10 | 5.85 | 17.45 | <0.01 |
| Sex (male/female) | | .047 | .018 | .12 | <0.01 |
| MNA | Malnutrition | 1 |  |  |  |
|  | At risk of malnutrition | .30 | .08 | 1.08 | 0.07 |
|  | Well nourished | .19 | .053 | .67 | 0.01 |
| Low Physical Activity | | 11.43 | 4.03 | 32.42 | <0.01 |
| Polypharmacy | | 1.69 | 1.12 | 2.56 | 0.01 |
| Multimorbidity | | 2.65 | 1.96 | 3.58 | <0.01 |

FI: frailty index, OR: odds ratio, CI: confidence interval
